# Preparation and Characterization of a Novel Salicin–Cyclodextrin Complex

**DOI:** 10.3390/pharmaceutics16030369

**Published:** 2024-03-06

**Authors:** Adina Căta, Ioana Maria Carmen Ienaşcu, Adina Frum, Daniel Ursu, Paula Svera, Corina Orha, Gerlinde Rusu, Adriana Aurelia Chiș, Carmen Maximiliana Dobrea, Claudiu Morgovan, Oana-Raluca Pop

**Affiliations:** 1 National Institute of Research and Development for Electrochemistry and Condensed Matter, 144 Dr. A. P. Podeanu, 300569 Timişoara, Romania; adina.cata@yahoo.com (A.C.); danielhoratiu@yahoo.com (D.U.); paulasvera@gmail.com (P.S.); orha.corina@gmail.com (C.O.); 2 Department of Pharmaceutical Sciences, Faculty of Pharmacy, “Vasile Goldiș” Western University of Arad, 86 Liviu Rebreanu, 310045 Arad, Romania; 3 Preclinical Department, Faculty of Medicine, “Lucian Blaga” University of Sibiu, 550169 Sibiu, Romania; adina.frum@ulbsibiu.ro (A.F.); adriana.chis@ulbsibiu.ro (A.A.C.); carmen.dobrea@ulbsibiu.ro (C.M.D.); claudiu.morgovan@ulbsibiu.ro (C.M.); 4 Faculty of Industrial Chemistry and Environmental Engineering, Politehnica University of Timișoara, 6 C. Telbisz, 300001 Timișoara, Romania; gerlinde.rusu@upt.ro; 5 Faculty of Pharmacy, University of Medicine and Pharmacy “Victor Babeș” Timișoara, 2 Eftimie Murgu Square, 300041 Timișoara, Romania; pop.raluca@umft.ro

**Keywords:** bioactive compounds, phytochemicals, anti-inflammatory, bioavailability, salicin, β-cyclodextrin

## Abstract

Bioactive compounds extracted from plants can provide wide health benefits. However, some molecules have limited applications as pharmaceuticals due to their limited solubility, poor bioavailability, and low stability when exposed to environmental factors. Their integration in formulations that can deliver them to physiological targets while preserving their biological activity can enhance their usage in improving human health. This research provides a delivery system design to enhance the solubility, stability and to mask the bitter taste of salicin. Thus, a novel salicin-β-cyclodextrin complex was prepared and analyzed by X-ray diffraction, scanning electron microscopy, thermogravimetric analysis, FTIR, Raman and UV-Vis spectroscopy. The analytical and computational methods provided clear and distinct evidence for inclusion of salicin within the β-cyclodextrin cavity and brought important findings for the characterization of the inclusion complex. The present study showed that salicin and β-cyclodextrin can form inclusion complexes, both in solution and in solid state, and that the inclusion of salicin in the cavity of β-cyclodextrin leads to the improvement of its solubility and stability. Thus, the study communicates both qualitative and quantitative knowledge about the preparation of a new salicin-β-cyclodextrin inclusion complex suggesting its potential applications in pharmaceutical industry and medical sciences, as formulations with better compliance for the patient, with increased bioavailability, and easier control of dosage.

## 1. Introduction

Plants represent sustainable sources of bioactive compounds and their valorization is of great concern whether they grow spontaneously or are cultivated. Worldwide the study of biologically active phytochemicals is of great interest due to their health benefits [1,2,3,4]. The use of natural compounds in the pharmaceutical industry, offers many advantages, such as higher biocompatibility and often lower side effects compared to synthetic ones [5,6,7,8]. To exert their biological activity, molecules must be able to reach their target without losing their integrity and to cross the lipophilic membranes. Although bioactive principles are abundant in plants, some molecules have limited applications as pharmaceuticals due to their limited solubility, poor bioavailability, being easily modified by environmental factors. Hence, to preserve the structural integrity of such bioactive molecules, they must be integrated in a formulation able to deliver them to physiological targets while preserving their biological activity [9,10]. Cyclodextrins can serve as vehicles for the protection of biologically active compounds due to their ability to encapsulate various molecules, from straight or branched aliphatic chains to polar compounds, forming inclusion complexes [10,11,12]. Cyclodextrins have the ability to improve the solubility of the guest molecule, to stabilize it against derivatizing agents thus increasing the shelf life and reducing the concentrations of the agent needed to achieve a biological effect [12,13,14,15]. In addition, cyclodextrins can modulate the delivery rate of the guest molecule [10,13,16], or they can eliminate the irritating or toxic effects of the active ingredient [10,11,12,16,17,18]. They can also change the taste by masking unwanted flavors, control odors [11,12,14,19], and are readily available at an affordable price.

The advantages of the complexation of some natural compounds are highlighted in the following examples. To increase the solubility in water and to improve its biological activity with respect to the free drug, morin (3,5,7,7,2′,4′-pentahydroxyflavone), a natural compound with antiangiogenic, antioxidant, anti-inflammatory, analgesic, and antitumoral activity was complexed with hydroxypropyl-β-cyclodextrin [20,21] and with sulfobutylether-β-cyclodextrin [20].

An inclusion complex containing puerarin, a bioactive isoflavone, and β-cyclodextrin was developed. The advantage of encapsulation consisted of higher solubility, faster dissolution rate and higher antioxidant capacity compared with that of uncomplexed puerarin [22].

Lupeol, a natural pentacyclic triterpene, with known anti-inflammatory and antitumor activity was complexed with hydroxy-propyl-gamma cyclodextrin and topically administered in a mouse model of photochemically induced skin carcinoma. The complexed lupeol showed an important reduction of the tumor mass and inflammatory reaction and also significantly improved the physiological parameters of the skin [23].

Some curcumin-cyclodextrins complexes were prepared to evaluate their anti-inflammatory activity in the treatment of inflammatory bowel disease using the colitis-induced rat model. Inclusion complexes increased the solubility of curcumin and the in vitro dissolution. The best results were obtained for HPβCD complex of curcumin, which was further investigated for its antiangiogenic and anti-inflammatory activity. HP-β-CD complex of curcumin proved to be a potent angio-inhibitory compound which significantly attenuated the degree of colitis caused by administration of dextran sulfate solution [24].

Although the most common and efficient anti-inflammatory agents against diseases accompanied by inflammation, pain and fever are synthetic drugs, they in many cases involve long-term therapy and also have a harmful impact on human health [25,26]. Therefore, finding natural compounds with minimal toxic side effects and good therapeutic effects to substitute traditional anti-inflammatory drugs is an imperative issue to be resolved in the clinical treatment of inflammation-related diseases, even if such natural compounds are more effective for relieving light pain in the early stage of these disorders [27]. Thus, for the treatment and prevention of arthritis, many researches have been conducted to explore the efficiency of herbal extracts and their bioactive compounds precisely to overcome the limitations of conventional synthetic drugs [28,29].

For example, willow bark is approved by the pharmacopeias and quality standards organizations all over the world, i.e., United States Pharmacopoeia (USP), European Pharmacopoeia (EP), European Medicines Agency (EMA), World Health Organization (WHO), and European Scientific Cooperative on Phytotherapy (ESCOP). A minimum of 1.5% of total salicylic derivatives, expressed as salicin, are stipulated in the corresponding monographs [30]. The European Pharmacopoeia commission approved willow bark extracts as a therapy for treating fever, rheumatic conditions, and headaches. The main compounds responsible for the activity of willow bark are salicin including its metabolites (e.g., salicylic acid), catechin, and flavonoids [31,32]. The recommended daily dose of salicin is 120 to 240 mg [30,31]. At this daily intake, willow bark extract could ensure an analgesic effect with less gastrointestinal side effects compared to acetylsalicylic acid or other nonsteroidal anti-inflammatory drugs [31,33].

Salicin, used as a biological activity marker of willow bark [34], is a white, bitter tasty powder [35]. Along anti-inflammatory properties, salicin proved to exhibit anti-irritative effect in topical dermatological applications [36], anti-aging property on the skin [37] and to prevent collagen type II from degradation by matrix metalloproteinases [38]. Recently, collagen films incorporated with salicin were prepared as new biomaterials with superior adhesion to the skin, that also benefit from the biological properties of salicin [39].

Different strategies could be useful to optimize the pharmaco-technic properties of salicin. In this respect, our research sought to obtain a novel salicin-β-cyclodextrin complex, which, in addition to improved safety, could ensure formulations with better compliance for the patient by masking the bitter taste of salicin, with a better bioavailability, and easier control of dosage. Thus, this research aimed to: (1) develop a novel inclusion complex salicin-β-cyclodextrin; (2) study the complexation in solution between salicin and β-cyclodextrin using UV-Vis spectroscopy; (3) obtain the salicin-β-cyclodextrin complex in solid state by kneading method; (4) demonstrate the formation of the inclusion complex, ensuring at the same time a complete characterization of it; (5) demonstrate the improvement of the solubility and stability of complexed salicin; and (6) offer both qualitative and quantitative knowledge about the inclusion complex of salicin and β-cyclodextrin, proving its application potential for pharmaceutical industries and medical sciences.

## 2. Materials and Methods

### 2.1. Chemicals

Reagents, salicin, β-cyclodextrin (Sigma-Aldrich, St. Louis, MO, USA, for synthesis) and solvent, absolute ethanol (Merck, Darmstadt, Germany, analytical purity), were used as purchased.

### 2.2. Apparatus

ATR-FTIR spectra were recorded on a Vertex 70 spectrophotometer equipped with ATR mode (diamond) (Bruker, Ettlingen, Germany), at room temperature, 128 scans, in the range 4000–450 cm^−1^. Raman spectra of the samples were obtained using the MultiView-2000 system (Nanonics Imaging Ltd., Jerusalem, Israel), equipped with a Shamrock 500i Spectrograph (ANDOR, Belfast, UK). The analysis was performed at room temperature through a 10× microscope objective using 514 nm laser excitation. X-ray powder diffraction (XRD) measurements were performed using an X’Pert PRO MPD apparatus (PANalytical, Almelo, The Netherlands), with a Cu Kα radiation (λ = 0.1541 nm). Scanning electron microscopy (SEM) analysis was performed using an Inspect S microscope (FEI, Eindhoven, Holland), at 30 kV, in vacuum mode, at 400–6000 magnification for all the samples. Thermogravimetric analysis (TGA) was carried out on a TG 209F1 Libra (Netzsch, Selb, Germany), in an inert medium, N_2_, 20 mL/min., with a heating rate of 10 K/min, in the temperature range 25–500 °C, and UV-Vis measurements on solid samples were carried out on a UV-Vis-NIR Lambda 950 (PerkinElmer, Waltham, MA, USA), in the range 200–1300 nm. Absorption spectra of samples for liquid phase complexation were recorded using a double beam JASCO V-530 UV-VIS spectrophotometer (ABL&E-JASCO, Wien, Austria) with 1 cm quartz cuvettes in the wavelength range 200–700 nm, at room temperature, against distilled water as a blank.

### 2.3. Liquid Phase Complexation

A stock solution of salicin in water was prepared at a concentration of 1 × 10^−3^ mol/L. 0.5 mL of the stock solution was added to β-cyclodextrin solutions of varying concentrations (1 × 10^−3^–9 × 10^−3^ mol/L), so the final concentration of the salicin in working samples was 1 × 10^−4^ mol/L. The obtained solutions were kept overnight at room temperature in order to reach equilibrium. Then, the absorption spectra of the inclusion complex formed in solution at different concentrations of β-cyclodextrin were recorded.

### 2.4. Obtaining the Inclusion Complex in Solid State

The inclusion complex of salicin and β-cyclodextrin was obtained using the kneading method. The two components, salicin and β-cyclodextrin at a 1:1 molar ratio, were ground in an agate mortar for 1 h, while continuously adding ethanol in order to obtain a paste. The obtained product was held in a heating chamber at 60 °C for 3 days and stored in a desiccator.

### 2.5. Obtaining the Physical Mixture

The physical mixture (PM) of salicin and β-cyclodextrin was prepared. First, the two components were individually ground in a mortar, then, at a molar ratio of 1:1, were mixed together till a homogenous mixture was obtained [40].

### 2.6. Solubility of Free and Complexed Salicin

The solubility of salicin in water was determined by the saturation solubility method [22]. An excess of salicin was added to the water and incubated in a 37 °C rocking shaker at 60 r/min for 24 h. After incubation, the solution was cooled at 25 °C and centrifuged at 3000× *g* for 15 min. The supernatant was filtered through a 0.45 μm PTFE membrane. The content of salicin was analyzed spectrophotometrically at 311 nm. The solubility of salicin in the inclusion complex was assessed by the same method.

### 2.7. Computational Details

The salicin structure was optimized at BLYP/TZP level of theory [41], implemented in ADF Modeling Suite 2014 [42]. Within the study, gas-phase and solution computations have been performed, using water (aq) and ethanol (EtOH) as solvents. The xyz coordinates of the optimized structures are provided in the Supplementary File S1. The graphical representation of the frontier molecular orbitals HOMO and LUMO has been also performed by means of ADF 2014. The geometric aspects including the dihedral angles, the partition coefficient logP and the steric parameters like ovality, solvent accessible area and solvent-excluded volume [43] were computed with Chem 3D (ChemBioDraw Ultra 14) software. The molecular docking computations have been performed with AutoDock Vina [44]. The same software has been employed for the visualization of the ligand-receptor interactions. β-cyclodextrin has been assigned as receptor, and a grid box of 40 × 40 × 40 Å was employed, the center of the grid box being considered the center of the cyclodextrin. The structure of the β-cyclodextrin has been downloaded from the Protein Data Bank (New Brunswick, NJ, USA) [45].

## 3. Results and Discussion

In order to improve the pharmaco-technic and pharmacological properties of salicin, this was incorporated in β-cyclodextrin, a natural, cheaper cyclodextrin, of which cavity is proper to encapsulate the molecule of salicin.

### 3.1. Stoichiometry and Association Constant Determination

In a first stage, the inclusion of the salicin in the cavity of β-cyclodextrin was studied in solution using UV-Vis spectroscopy, a method that can be used to prove the formation of the inclusion compounds [46,47]. 

**Figure 1:**
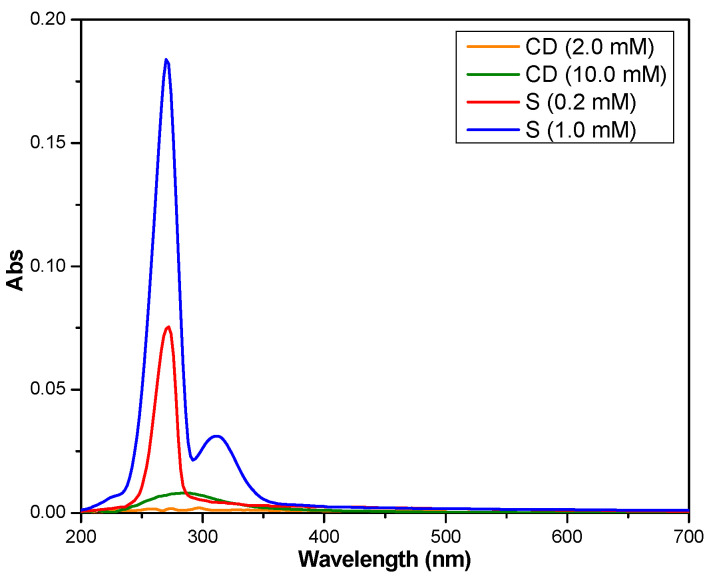
Absorption spectra of β-cyclodextrin and salicin at different concentrations.

**Figure 2:**
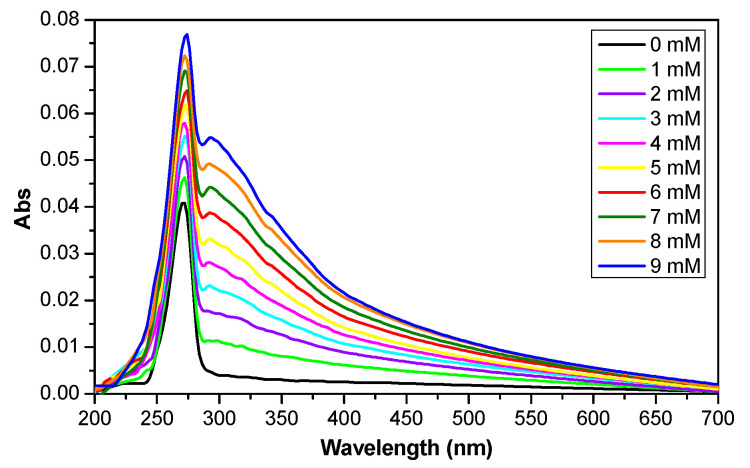
Absorption spectra of salicin with various concentrations of β-cyclodextrin.

Figure 1 shows the absorption spectra of both salicin (0.2 mM and 1 mM) and β-cyclodextrin (2.0 mM and 10 mM). Salicin has two absorption bands, at 272 nm and 311 nm, attributed to the π → π* transitions in disubstituted benzene, the latter seems that it is due to the local excitation of phenyl rings [48]. The band at 311 nm is visible at concentrations higher than 0.2 mM. As for the β-cyclodextrin, this shows a very weak absorbance at 286 nm that can be neglected. Thus, it can be considered that the absorbance of the inclusion compound (Figure 2) is due to the salicin alone. 

The behavior of salicin in the presence of increasing β-cyclodextrin concentrations was established based on the UV-Vis absorption spectra of such mixtures. With the increase in cyclodextrin concentration, an increase in absorbance could be observed (Figure 2). This finding can be attributed to the appearance of a nonpolar environment around the guest molecule upon forming the complex [48], proving the formation of the inclusion complex between the salicin and β-cyclodextrin.

On the other hand, salicin will probably be accommodating in the hydrophobic cavity of β-cyclodextrin with benzene part also, so the stoichiometric ratio of the inclusion complex should theoretically be 1:1. A proof of this inclusion ratio can be obtained when a linear relationship is obtained from the reciprocal plot of 1/∆*A* vs. 1/[*CD*] based on the Benesi-Hildebrand Equation (1) [40].

(1) $$\frac{1}{∆A}=\frac{1}{∆\varepsilon {\left[G\right]}_{0}K\left[\mathrm{CD}\right]}+\frac{1}{∆\varepsilon {\left[G\right]}_{0}}$$

where

− ∆*A* is the difference between the absorbance of the complex and the absorbance of the salicin;
− ∆*ε* is the difference between the molar absorptivity of the complex and the molar absorptivity of the salicin;
− [*G*]_0_, *K*, [*CD*], are the initial concentration of salicin, the apparent formation constant of the inclusion complex and the concentration of β-cyclodextrin, respectively.

**Figure 3:**
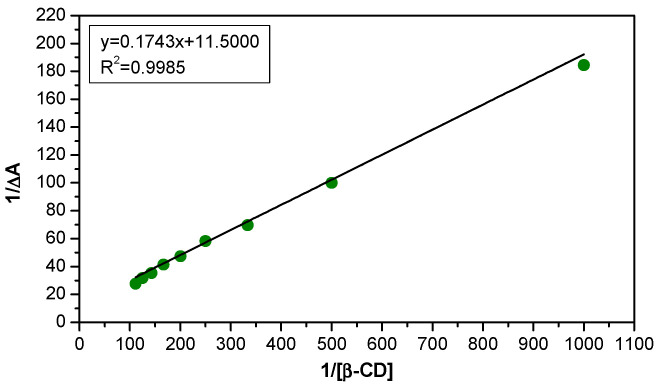
The Benesi–Hildebrand plot for the complexation of the salicin and β-cyclodextrin at 272 nm.

Figure 3 represents the Benesi-Hildebrand plot for the complexation of the salicin and β-cyclodextrin at 272 nm. A good linear relationship was obtained for 1/∆*A* vs. 1/[*CD*], with R^2^ = 0.9985 (Figure 3), from which it can be deduced that the stoichiometry ratio for the inclusion complex between salicin and β-cyclodextrin is 1:1. Similar phenomena were observed in other researches [40,47].

The apparent formation constant of the inclusion complex, *K*, the variation of the molar absorptivity, ∆*ε*, and the molar absorptivity of the IC, *ε*_IC_, were established in order to characterize the inclusion complex in liquid phase. 

The parameters *K* and ∆*ε* were calculated from the Benesi–Hildebrand plot (Figure 3), according to Equations (2) and (3) [40].

(2) $$K=\frac{{(y}_{intercept})}{\left(\mathrm{slope}\right)}$$

(3) $$∆\varepsilon =\frac{1}{{\left[G\right]}_{0}(y_{intercept})}$$

So, after the calculations, the values of *K* and ∆*ε* were established as 65.987 ± 14.091 M^−1^ and 869.564 ± 183.369 L·mol^−1^·cm^−1^, respectively.

The inclusion complex formation constant, K, also known as equilibrium constant or binding constant, is a parameter that represents the thermodynamic equilibrium between the free and the complexed molecules [10] and allow to determine the thermodynamic parameters associated to the complex formation [49]. Many authors choose to characterize the inclusion complexes in solution using the binding constant obtained from Benesi-Hildebrand plots. For instance, [49] found for 7-amino-3-phenyl-2H-benzo[b][1,4]oxazin-2-one in aqueous solutions of β-cyclodextrin using steady-state fluorescence, a value of 597 M^−1^ at 298 K. The study also showed that the K value is influenced on pH due to the changes in molecule charge [49]. Malapert et al. studied the complexation between β-cyclodextrin and hydroxytyrosol in solution [50]. Based on the K value, 33.2 ± 3.7 M^−1^, the percentage of complex in solution from an equimolar (5 mM) mixture of both partners was established (lower than 20%) [50]. Eteer et al. determined for the vanillin-β-CD system the binding constant of 179 M^−1^ [51]. Prabu, et al. investigated the behavior of the inclusion of guanosine in β-cyclodextrin in the liquid state obtaining a value of 59.79 M^−1^ at 303 K for the binding of the inclusion complex [52].

**Figure 4:**
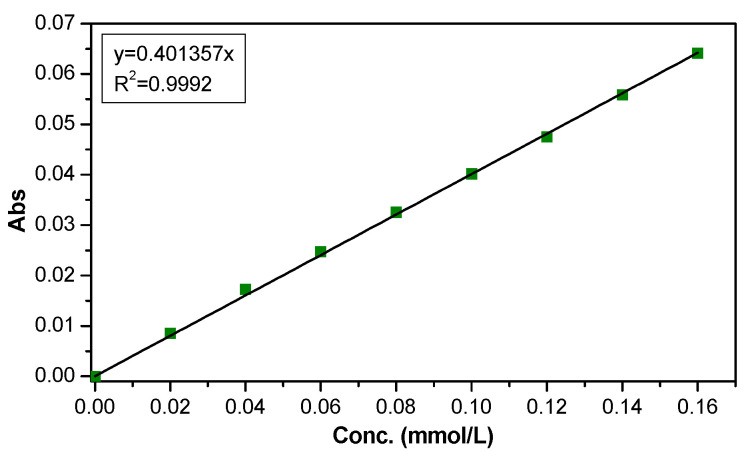
The calibration curve of the salicin at 272 nm.

The molar absorptivity of the IC (*ε*_IC_) can be calculated according to Equation (4), where *ε*_S_ is the molar absorptivity of salicin and was determined using the calibration curve of salicin (Figure 4).

∆*ε* = *ε*_IC_ − *ε*_S_ (4)

Thus, the value of parameter *ε*_S_ was determined as 401.357 L·mol^−1^·cm^−1^ and *ε*_IC_ was calculated as 1270.92 L·mol^−1^·cm^−1^.

### 3.2. Characterization of the Solid State Inclusion Complex

**Scheme 1:**
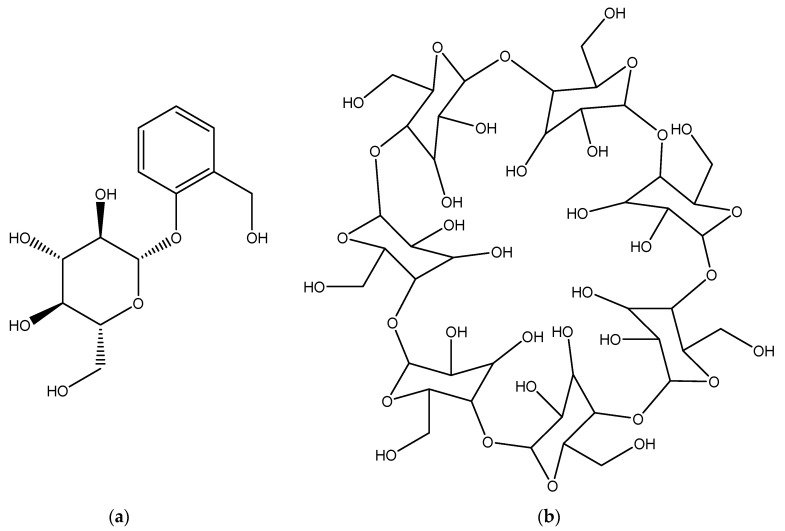
Structures of the two components of the salicin-β-cyclodextrin complex: (**a**) salicin; (**b**) β-cyclodextrin.

In the second stage, the obtaining of the inclusion complex between salicin and β-cyclodextrin was studied in solid phase. The molecule of salicin (Scheme 1a) contains both hydrophobic and hydrophilic parts. Thus, the benzene fragment of salicin, being hydrophobic, can be accommodated in the central cavity of β-cyclodextrin (Scheme 1b), also hydrophobic.

The salicin-β-cyclodextrin complex (IC) obtained by kneading method is a white powder which was further analyzed together with the physical mixture (PM) in order to better understand the formation of the inclusion complex. Inclusion complex characterization was accomplished based on XRD, SEM, TGA, FTIR, Raman and UV-Vis spectroscopy.

**Figure 5:**
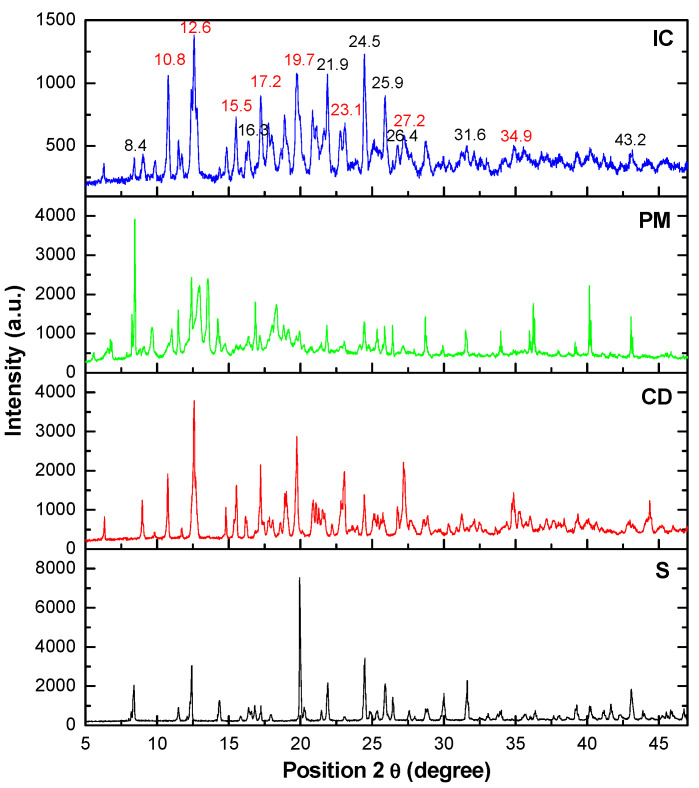
XRD spectra of salicin (S), β-cyclodextrin (CD), physical mixture (PM), and inclusion complex (IC).

Figure 5 presents the X-ray diffraction spectra of the β-cyclodextrin, salicin, the physical mixture and inclusion complex. 

The X-ray diffractograms (Figure 5) were used to study the change in crystal morphology of the parent crystalline molecules when converted into inclusion compounds. Both salicin (S) and β-cyclodextrin (CD) are crystalline compounds, with narrow and intense peaks in diffractograms. The physical mixture (PM) keeps the crystalline character of both its components, meanwhile the inclusion complex (IC) reveals a more amorphous nature. The XRD spectra of inclusion complex (IC) reveals a drastic reduction in intensity of some spectral lines corresponding to salicin (8.4°, 16.8°, 25.3°, 26.4°, 30.0°, 31.6°, 43.1°) and β-cyclodextrin (10.7°, 12.5°, 17.2°, 19.7°, 23.0°, 24.4°, 27.1°). Thus, due to the complexation phenomenon, the encapsulated product exhibits a broadening of the diffraction peaks meaning a lower degree of crystallinity, which confirm the inclusion and the amorphous nature of the complex [53,54]. 

**Figure 6:**
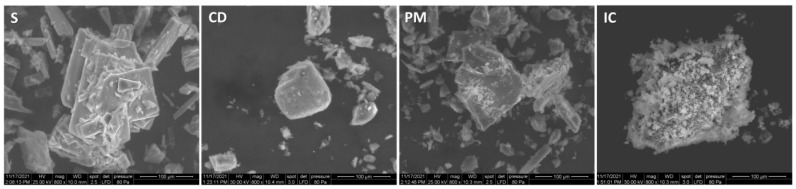
SEM images of salicin (S), β-cyclodextrin (CD), physical mixture (PM), and inclusion complex (IC).

The decreased in crystallinity in case of the inclusion complex was also proved by the SEM images (Figure 6). Figure 6 illustrates the surface morphology for the pure crystalline components (S and CD) and the prepared binary mixtures (physical mixture—PM and inclusion complex—IC).

In the case of the physical mixture (PM), the original morphology of both pure components used is found. SEM images of the inclusion complex (IC) show the presence of micrometric formations with irregular shapes indicating a more pronounced amorphous character of the product.

**Figure 7:**
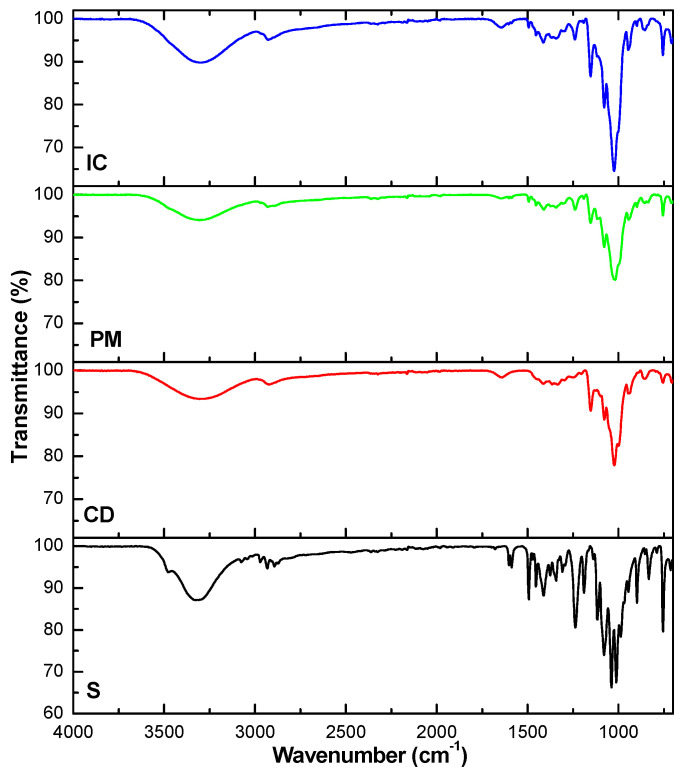
FTIR spectra of salicin (S), β-cyclodextrin (CD), physical mixture (PM), and inclusion complex (IC).

IR spectroscopy represents a useful tool to elucidate the interaction between host and guest molecules in the structure of inclusion complexes. ATR-FTIR spectra of the salicin, β-cyclodextrin, physical mixture, and inclusion complex are presented in Figure 7. 

The spectra recorded for the physical mixture (PM) and for the inclusion complex (IC) show similar characteristics to that of β-cyclodextrin (CD), with shifts of some cyclodextrin-specific bands to higher frequencies in the IC. For example, the CD band at 2923.94 cm^−1^ corresponding to the stretching vibrations ν[CH_2_] is shifted to 2927.80 cm^−1^ in the IC, and the one that corresponds to the bending vibrations ν[O–H] is shifted from 1022.22 to 1024.15 cm^−1^ (IC). Enhancement of the intensity of the broad peak at around 3305.83 cm^−1^, proved the increase in the number of hydroxyl groups and the complexation phenomena [53]. As for the salicin, the bands at 1602.77 and 1587.34 cm^−1^, which can be attributed to the skeleton vibration of benzene nucleus, move towards 1604.70 and 1589.34 cm^−1^, respectively. Similarly, the band assignable to glycosidic ν[C–O–C] shifts from 1238.24 to 1240.17 cm^−1^. The increase in frequency can be attributed to the increase in electron cloud density simultaneously with the inclusion of the benzene nucleus inside the β-cyclodextrin cavity [54,55]. Also, the decrease in intensity of the specific bands of salicin in the IC can be ascertained. All these modifications confirm the formation of the inclusion complex. Moreover, in the spectra of the inclusion complex, no new bands are present proving that no chemical bonds were formed between the guest and the host in the inclusion complex.

The advances in Raman spectroscopy promote this tool for several applications in the pharmaceutical industry. Thus, Raman spectroscopy can probe the formation of the inclusion complexes, providing fresh insights into the type of interactions present in such combinations [56]. Since β-cyclodextrin is a heptasaccharide derived from glucose it is useful to start looking for the active bands that are present also in the base compound. Thus, the glucose Raman data from the literature, reveal a highest wavenumber region 3000–3800 cm^−1^, linked to the vibrational frequencies of the stretching motions (ν) of monosaccharides hydroxyl groups [57,58,59]. CH_2_ stretching vibrations (ν) are found in the 2800–3000 cm^−1^ region [57,58,59]. Bending vibrations (δ) specific for CH_2_ and COH are depicted in the region 1200 to 1500 cm^−1^ and are related to the exocyclic conformation vibrations of the CH_2_OH group [57,58,59]. The 950–1200 cm^−1^ region highlights the deformation vibration (δ) of C-OH, C-CH and O-CH groups characteristic for ring conformation [57,58,59]. The region between 950–700 cm^−1^ is specific for the COH, CCH and OCH side-groups deformations and stretching vibrations of C-C [58]. Region bellows 700 cm^−1^ is referring to the skeletal region, more precisely, between 700–500 cm^−1^ the exocyclic deformations (CCO) are taking place, whereas below 500 cm^−1^, the endocyclic (CCO, CCC) [58]. Below the 200 cm^−1^ molecular interactions are revealed (hydrogen bonding, inter-crystalline forces) [58]. Other oligosaccharide that resembles the cyclodextrins is maltotetraose from clase of maltodextrin, consisting of four glucose molecules linked with α-1,4 glycosidic bonds. Mrozek et al. made a comparison between two oligosaccharides, maltotetraose and stachyose, which have identical mass but slightly different structure. From the Raman analysis, bonds are expected to be present in the range 1600 and 300 cm^−1^, more precisely HCH and CH_2_OH vibrations (1500–1200 cm^−1^), C–O stretching vibrations (1200–950 cm^−1^), side-group deformations (950–700 cm^−1^), and skeletal deformations (below 700 cm^−1^) [57]. The spectra for maltotetraose resembles very much those of β-cyclodextrin obtained in our case.

**Figure 8:**
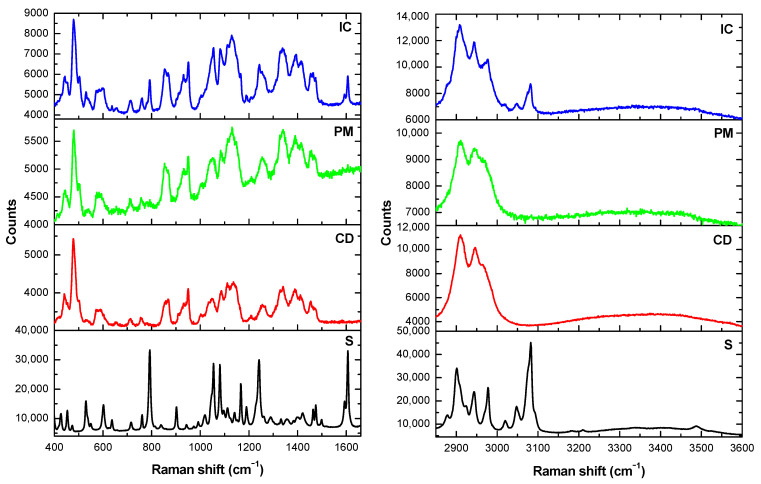
Raman spectra of salicin (S), β-cyclodextrin (CD), physical mixture (PM), and inclusion complex (IC).

Figure 8 shows the Raman spectra of the guest (S) and host (CD) as such, and that of the β-cyclodextrin, physical mixture (PM) and inclusion complex (IC).

After analyzing the Raman spectra (Figure 8), visible changes were observed when compared the samples. In the 400–1600 cm^−1^ region the endocyclic changes are visible. As found in literature, a very sharp and intense peak specific for α(1-4) glycosidic link vibrations is observed around 478 cm^−1^, which is present in oligo- and polysaccharides, but absent in glucose [59]. This signal appears in our case in the spectra of β-cyclodextrin, physical mixture and inclusion complex, being absent in salicin spectra. Specific α-anomeric configuration in case of β-cyclodextrin is present at 711, 753, and 860 cm^−1^ which are slightly shifted according to the literature [59]. Another important peak is located at 950 cm^−1^ indicating glycosidic COC stretching [59]. The peaks between 950–1200 cm^−1^ in the spectra of salicin, characteristic for the simple glucose [59], can also be found in the spectra of the inclusion complex. CH_2_OH deformations are assigned to 1338 cm^−1^ [59]. The peak at 796 cm^−1^ in the spectra of salicin can be assigned to the aromatic ring breathing mode, and the one at 1233 cm^−1^ to C-CH_2_OH stretching frequency, both of them visible in the spectra of inclusion compound, but decreased in intensity. Due to the lack of signals in the region 1600–1700 cm^−1^ of the Raman spectra of cyclodextrins, remains a free space for monitoring relevant guest modes [56], in our case, aromatic bonds. These appear at 1601 cm^−1^ as a strong intensity peak in the spectra of salicin and as low intensity peak in the inclusion complex. The decrease in intensity of this signal in the Raman spectra of the inclusion complex is apparently due to shielding of the benzene ring under the influence of the cyclodextrin cavity, that acts like a cage, and may limit the aromatic vibrations in Raman spectra [56]. This is clear evidence that the aromatic ring of the salicin moiety has been included in the cyclodextrin cavity. In the region 3200–3600 cm^−1^ wide-shaped peak, resembling a shoulder, specific for O-H stretching vibrations was detected in case of all compounds. In addition, C-H stretching band was observed between 2800–3100, more precisely at 2911 and 2943 cm^−1^ in case of β-cyclodextrin and physical mixture, whereas in case of salicin several peaks were observed. In case of the inclusion complex, the Raman spectra revealed the specific β-cyclodextrin peaks and three other located at 2979, 3046 and 3082 cm^−1^ which were also found in salicin spectra and correspond to aromatic bonds.

From the results it is clear that the inclusion compound was formed, with the existing bonds from both guest and host materials. When combined just physically, there is a higher fluorescence, but only cyclodextrin bands are present. Since it is the bigger molecule in the mixture, there is a probability that the laser might have been focused on certain molecule, still the traces of the other compound do not appear, suggesting that there is no bond between the two of them.

**Figure 9:**
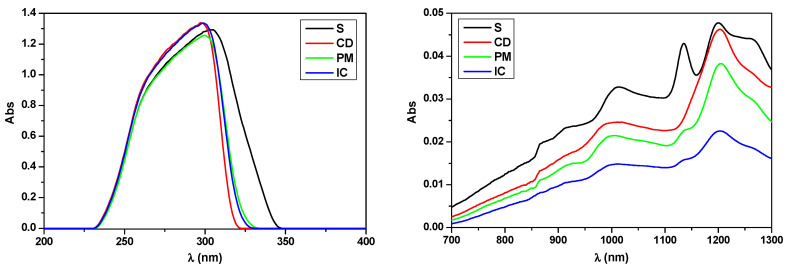
UV-Vis-NIR spectra of salicin (S), β-cyclodextrin (CD), physical mixture (PM), and inclusion complex (IC).

Another important tool used to prove the formation of the inclusion compounds is UV-Vis spectroscopy. The UV-Vis spectra of the samples are depicted in Figure 9. The absorption maxima in the UV region (Figure 9, left) of the host and guest compounds occur at fairly close wavelengths, 297 nm for cyclodextrin and 302 nm for salicin. The UV-Vis spectra for the binary compound show an absorption maximum at 298 nm, located between the maxima of the pure components, the profile being very similar to that of β-cyclodextrin. Significant spectral differences between the pure components and the physical mixture can be observed in the NIR region (Figure 9, right), which may suggest the formation of the inclusion complex.

**Figure 10:**
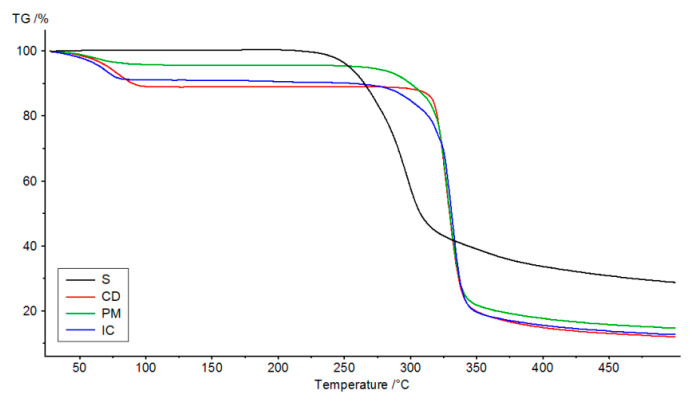
TGA curves of salicin (S), β-cyclodextrin (CD), physical mixture (PM), and inclusion complex (IC).

Thermal analysis is one of the most frequently instrumental techniques used to characterize the thermal stability and decomposition of active ingredients [60]. In the present study, the thermal degradation of β-cyclodextrin (CD) shows three stages during the heating process of the sample (Figure 10). The first stage, from room temperature to 100 °C, corresponds to the loss of water (11.05% of total weight of CD) coming from the desorption of moisture as bound water on the surface and from the cavity of CD [61]. The second stage of degradation, characterized by major weight loss, goes from 292 to 390 °C and can be assigned to the main pyrolysis process of the β-cyclodextrin [61]. In the temperature range 390–500 °C (third stage), β-cyclodextrin continuously decomposed at a very slow rate, so, at the final temperature of the experiment (500 °C), the residual β-cyclodextrin represents 12.06% of initial weight. On the other hand, salicin shows a single loss of mass in the temperature range 202–357 °C, due to the degradation of the benzene nucleus present in its structure. The lower limit of the temperature range related to the decomposition of salicin corresponds to the melting point of salicin (202 °C).

Regarding the behavior of the inclusion complex (IC), it can be seen that the temperature of degradation shifted from 297.4 °C (inflection point for salicin) and 326.0 °C (inflection point for β-cyclodextrin) to 331.3 °C (inflection point for inclusion complex), proving the increase of thermal stability in case of encapsulated salicin. Moreover, the physical mixture (328.2 °C) revealed also a greater stability than the neat components, suggesting the benefits of the β-cyclodextrin presence on the stability of salicin. All these phenomena show that the formation of the inclusion complex altered the thermal degradation properties of the two components as such [54].

Furthermore, as Belyakova et al. stated in case of salicylic acid-β-cyclodextrin complex, the increased thermal stability of the inclusion complex in comparison to salicylic acid indirectly prove the absence of strong specific interactions, i.e., hydrogen bonding, between host and guest molecules [48], which can be seen in our case as well. Besides, the interaction of salicin with β-cyclodextrin leads to the formation of the inclusion complex probably through the dispersion attraction of salicin aromatic ring to the internal cavity of the β-cyclodextrin along the formation of weak hydrogen bonds between phenyl hydroxyls of salicin and glycoside oxygens of β-cyclodextrin. This phenomenon, noticed by other researchers [48], is in accordance with the computational data regarding the ligand-receptor interactions (Section 3.4). 

### 3.3. Evaluation of the Complexation Effect on Salicin Solubility

The solubility in water of free and complexed salicin is shown in Table 1. After the inclusion in β-cyclodextrin, the solubility of salicin was 85.80 g/L, which was 2 times higher than that of the free salicin (43.19 g/L). 

The inclusion in β-cyclodextrin enhanced the solubility of salicin in water. Along with increased solubility, irritation, stability and bitter taste could be significantly improved since the surface physicochemical properties of the molecule are masked [22,62]. The solubility experiment shows that the complexation with β-cyclodextrin has a significant effect on improving the solubility of salicin and it is possible that a larger amount of salicin could reach the target, and thus the bioavailability of salicin could be enhanced.

### 3.4. Molecular Modeling

**Figure 11:**
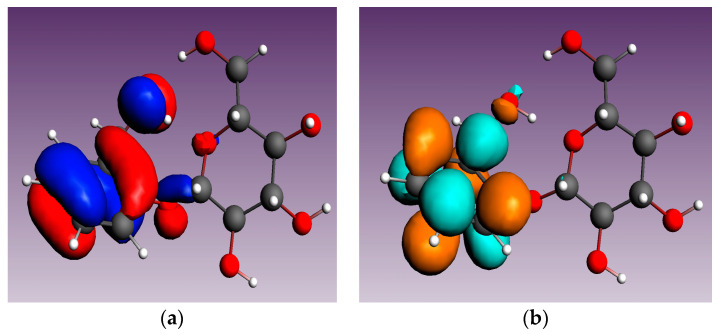
The frontier molecular orbitals of gas-phase optimized salicin: (**a**) HOMO orbitals; (**b**) LUMO orbitals.

Concerning the gas-phase, the graphical representation of the frontier molecular orbitals of salicin shows that both the HOMO and LUMO orbitals are found on the glucoside ring, including its OH groups (Figure 11). 

**Figure 12:**
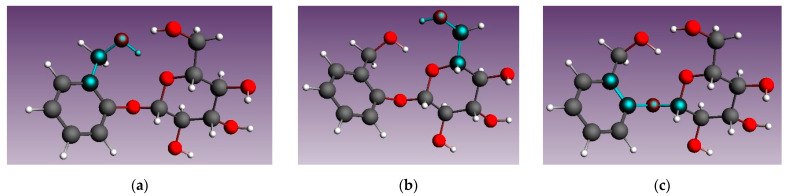
The investigated dihedral angles of the gas-phase, aqueous and ethanol solvated salicin (the corresponding atoms are highlighted in green): (**a**) dihedral angle (1); (**b**) dihedral angle (2); (**c**) dihedral angle (3).

A comparison of the optimized salicin structures has been performed (Figure 12), in order to evaluate the possible influence of the solvent. Three different dihedral angles have been compared, reflecting the position of the hydroxymethyl groups from the phenyl and glycoside residues (dihedral angles 1 and 2) and of the phenyl and glycoside moieties (dihedral angle 3). Figure 12 illustrates the investigated dihedral angles.

**Figure 13:**
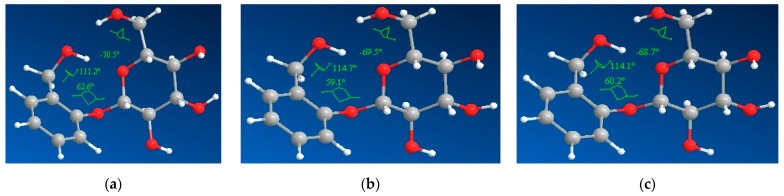
Optimized structures of the gas-phase, aqueous and ethanolic salicin: (**a**) gas; (**b**) aqueous; (**c**) ethanolic.

The calculated angles of the solvated structures (Figure 13) show similar values, while the largest differences when compared to the gas-phase structure have been obtained for the dihedral angles (1) and (3) (111° vs. 114°, and 62° vs. 59°, respectively). The calculated angles of the solvated structures are depicted in Figure 13. 

According to the above-mentioned results, the steric parameters of gas-phase and solvated-optimized salicin show no significant differences; the results are depicted in Table 2.

The results of the molecular docking study are included in Table 3.

**Figure 14:**
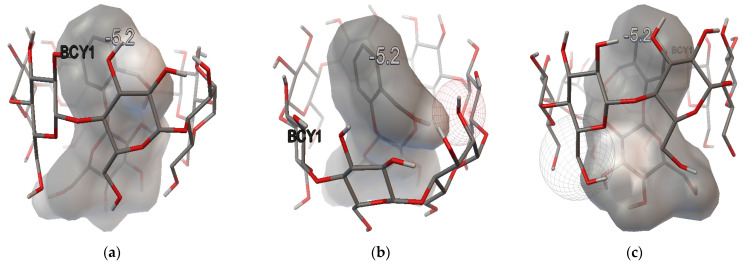
Structure of salicin-β-cyclodextrin complexes: (**a**) salicin gas; (**b**) salicin aqueous; (**c**) salicin ethanolic.

**Figure 15:**
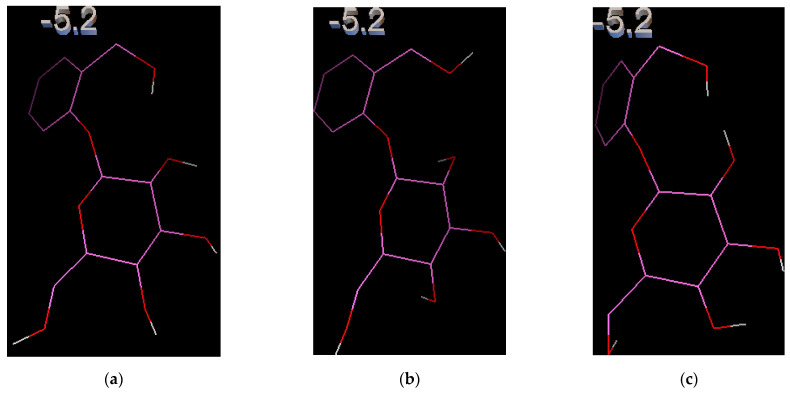
The best salicin conformations in interactions with β-cyclodextrin: (**a**) salicin (gas); (**b**) salicin (aq); (**c**) salicin (EtOH).

The size of β-cyclodextrin allows the inclusion of the salicin in its cavity (Figure 14). As shown in Table 3, the best binding affinities were obtained for salicin in gas-phase (−4.97 kcal/mol). When using other solvents, i.e., water and ethanol, it seems that the latter confers a more stable conformation of the inclusion complex (−4.90 kcal/mol), although the values for binding energy are quite similar. The structure of the salicin-β-cyclodextrin interactions is depicted in Figure 14:

The best ligand conformations of salicin in interactions with β-cyclodextrin are depicted in Figure 15:

The interaction of gas-phase and solvated salicin with β-cyclodextrin is presented in Table 4.

Regarding the interaction of gas-phase and solvated salicin with β-cyclodextrin (Table 4), in all three cases, atoms in close contact are present, only in case of aqueous salicin one hydrogen bond is formed, that suggests the inclusion of the salicin within the β-CD structure.

The values of the three dihedral angles reflecting the position of the hydroxymethyl groups from the phenyl and glycoside residues (dihedral angles 1 and 2) and of the phenyl and glycoside moieties (dihedral angle 3) are presented in Table 5:

Compared to the optimized structures (see Figure 13), the main differences have been obtained for the dihedral angles 1 and 2, which are correlated with the position of the hydroxymethyl groups from the phenyl and glycoside residues. It may be observed the position of the hydroxymethyl group for salicin (aq), which allows the formation of a hydrogen bond with β-cyclodextrin.

## 4. Conclusions

This research provides a delivery system design to enhance the solubility, stability and to possibly mask the bitter taste of salicin. The value of the association constant of the salicin-β-cyclodextrin inclusion complex in aqueous solution, 65.987 M^−1^, was determined based on the Benesi-Hildebrand equation. All the analytical methods used provided clear and distinct evidence for inclusion of salicin within the β-cyclodextrin cavity and brought important findings for the characterization of the inclusion complex. Thus, the encapsulation of salicin changes its thermal stability and crystal structure, leading to a more pronounced amorphous character of the inclusion complex. By complexation, the solubility of salicin was increased to a double value compared to free salicin. The absence of strong specific interactions between host and guest molecules was also proved, with salicin stabilized in the cavity of β-cyclodextrin by means of dispersion attraction and weak hydrogen bonds. Thus, the study communicates both qualitative and quantitative knowledge about the formation of the inclusion complex of salicin with β-cyclodextrin suggesting its potential applications in pharmaceutical industries and medical sciences.

## Figures and Tables

**Table 1:** The solubility of free and complexed salicin (mean ± SD, n = 3).

Compound	Solubility (g/L)
salicin (free)	43.19 ± 1.06
salicin (complexed)	85.80 ± 1.26

**Table 2:** The calculated steric parameters of salicin.

Compound	Connolly Accessible Area (Å2)	Connolly Solvent-Excluded Volume (Å3)	Ovality	logP
Salicin (gas)	483.013	245.948	1.344	−0.471
Salicin (aq)	486.480	247.799	1.345	
Salicin (EtOH)	485.620	247.613	1.343	

**Table 3:** The final Lamarckian genetic algorithm docked state—best binding affinities of salicin with the β-cyclodextrin (kcal/mol).

Compound	E (kcal/mol)									
	E1	E2	E3	E4	E5	E6	E7	E8	E9	E
Salicin (gas)	−5.2	−5.1	−5.1	−5.0	−4.9	−4.9	−4.9	−4.8	−4.8	−4.97
Salicin (aq)	−5.2	−5.0	−4.9	−4.9	−4.9	−4.8	−4.8	−4.7	−4.7	−4.88
Salicin (EtOH)	−5.2	−5.0	−4.9	−4.9	−4.9	−4.8	−4.8	−4.8	−4.8	−4.90

**Table 4:** Analysis of the ligand-receptor interactions between salicin and β-cyclodextrin.

Compound	Interactions with β-Cyclodextrin
Salicin (gas-phase)	Atoms in close contact
Salicin (aq)	Atoms in close contact 1 hydrogen bond (1.981 Å) (phenyl OH–CD)
Salicin (EtOH)	Atoms in close contact

**Table 5:** Dihedral angles characterizing the best ligand structure of salicin.

Best Ligand Conformation	Dihedral Angle 1 (°)	Dihedral Angle 2 (°)	Dihedral Angle 3 (°)
Salicin (gas)	−65.9	−153.1	−130.9
Salicin (aq)	160.8	167.9	−137.2
Salicin (EtOH)	−1.7	−32.3	64.3

## Data Availability

The data presented in this study are available in this article and Supplementary Material.

## Supplementary Materials

The following supporting information can be downloaded at: https://www.mdpi.com/article/10.3390/pharmaceutics16030369/s1, File S1: The xyz coordinates of the three optimized structures of salicin.
