# Supplementary material for: Preparation and Characterization of a Novel Salicin–Cyclodextrin Complex
Source: Pharmaceutics. 2024 Mar 6;16(3):369. doi: 10.3390/pharmaceutics16030369 (PMC10976097; doi:10.3390/pharmaceutics16030369)
Supplement: Supplementary file 1 [file pharmaceutics-16-00369-s001.zip › pharmaceutics-2853102-supplementary.pdf]

File S1: The xyz coordinates of the three optimized structures of salicin.

xyz coordinates of salicin (gas)

O	0.63341206	0.44755225	-0.61642907
O	-0.78611203	-1.35403160	-0.46576106
O	3.97547676	-1.81188224	0.13644241
O	4.16621880	1.11287896	0.38840547
O	1.36282464	-2.89426962	0.72533374
O	0.54425167	3.38862798	-0.53371461
O	-1.60925721	1.41189114	-1.96007696
C	2.84727084	-0.95742346	0.42658424
C	3.01812114	0.44356794	-0.19896449
C	1.58440133	-1.70452924	-0.05593318
C	1.71036408	1.25565456	-0.06415625
C	0.36761129	-0.78233473	0.11258881
C	1.69921688	2.58704718	-0.83057755
C	-2.01820553	-0.99976502	0.14248906
C	-2.92482302	-0.18353842	-0.56346574
C	-2.32526814	-1.52533390	1.40233924
C	-4.15176965	0.10622632	0.06036188
C	-2.61287750	0.36101873	-1.95028219
C	-3.55363526	-1.21646939	2.00031689
C	-4.46905196	-0.39663265	1.32830355
H	2.75576230	-0.83360725	1.52360035
H	3.27235379	0.33503049	-1.26139523
H	1.68586815	-1.95013128	-1.12534404
H	1.49725082	1.45997122	1.00190907
H	0.19529189	-0.54570462	1.17548325
H	1.78069462	2.38000508	-1.91045636
H	2.57257890	3.17522730	-0.52761858
H	4.78017712	-1.30517657	0.36797048
H	3.95757766	1.30893767	1.32453191
H	2.19675005	-3.40459560	0.69683597
H	-0.24658636	2.96629040	-0.93105921
H	-1.61052824	-2.18460682	1.89244438
H	-4.86714688	0.74069467	-0.46334275
H	-2.29323672	-0.45921890	-2.61149564
H	-3.51889288	0.80810186	-2.37377733
H	-3.79523734	-1.62560743	2.98037555
H	-5.42670787	-0.15305921	1.78632814
H	-0.78397117	0.99820021	-1.61981764

xyz coordinates of salicin (aq)

O	0.58928407	0.46180314	-0.52617793
O	-0.78347643	-1.38942450	-0.39352983
O	3.99723854	-1.80260552	-0.02497822
O	4.14446204	1.13413086	0.41336987
O	1.42025832	-2.95464931	0.56532555
O	0.48980737	3.38917686	-0.36367705
O	-1.61210601	1.24259798	-2.11195485
C	2.87402288	-0.97562076	0.37071569
C	3.00490609	0.46323079	-0.18136651
C	1.60273170	-1.69581502	-0.12322013
C	1.68835553	1.25128310	0.03212510
C	0.38555464	-0.80427970	0.15678682
C	1.66885802	2.60448402	-0.68387324
C	-2.01228713	-0.97487228	0.18799832
C	-2.92053544	-0.24762290	-0.60625820
C	-2.31632279	-1.34957830	1.50071384
C	-4.15469380	0.10287954	-0.02769312
C	-2.59000150	0.15205627	-2.03306983
C	-3.54887818	-0.97954197	2.05582327
C	-4.47076293	-0.25191146	1.29036829
H	2.83178989	-0.92466412	1.47134144
H	3.22884378	0.41565043	-1.25448931
H	1.66876096	-1.86126136	-1.20975604
H	1.50688598	1.40077524	1.10886349
H	0.26072763	-0.61838028	1.23371725
H	1.74626647	2.45026490	-1.76992019
H	2.52780573	3.19459175	-0.35207714
H	4.78804289	-1.45765521	0.43722830
H	3.99379674	1.19685289	1.37993405
H	2.26783024	-3.43947841	0.48373826
H	-0.28612327	2.95362056	-0.77269832
H	-1.59991269	-1.93187228	2.07766763
H	-4.87289210	0.66426613	-0.62519295
H	-2.21356805	-0.71072796	-2.59929722
H	-3.49128220	0.52552204	-2.52777843
H	-3.78753268	-1.26940122	3.07809081
H	-5.43140692	0.03431858	1.71593098
H	-0.80125318	0.91790252	-1.65323652

xyz coordinates of salicin (EtOH)

O	0.59608261	0.45887941	-0.53423646
O	-0.76899135	-1.39426828	-0.39786182
O	4.00844019	-1.76947861	0.09408346
O	4.13778142	1.16815007	0.42608008
O	1.42585488	-2.92791893	0.64407050
O	0.46558704	3.37949231	-0.44264937
O	-1.59206477	1.23449890	-2.12675815
C	2.86953407	-0.94115959	0.43432089
C	3.00382140	0.48019193	-0.15895129
C	1.61499043	-1.68446628	-0.06908570
C	1.68256698	1.26711517	0.02038122
C	0.38576207	-0.79589196	0.16672450
C	1.65973453	2.60461884	-0.72452595
C	-2.00935695	-0.99070178	0.16688278
C	-2.91200826	-0.26323341	-0.63383437
C	-2.32851090	-1.37846321	1.47247619
C	-4.15640191	0.07255638	-0.06789582
C	-2.57079236	0.14600612	-2.05609688
C	-3.57115206	-1.02332666	2.01435067
C	-4.48733238	-0.29482791	1.24301292
H	2.79851585	-0.85585630	1.53193400
H	3.23701629	0.40190495	-1.22829257
H	1.70445654	-1.87343227	-1.15039660
H	1.48676173	1.43930221	1.09152100
H	0.23669867	-0.59333335	1.23819078
H	1.75939552	2.42826182	-1.80564127
H	2.50547655	3.21239713	-0.39013228
H	4.79504964	-1.36221313	0.51137833
H	3.97320195	1.26997800	1.38712837
H	2.27273061	-3.41621938	0.57942438
H	-0.29976034	2.91856001	-0.84459969
H	-1.61532157	-1.96230241	2.05188602
H	-4.87208568	0.63301046	-0.66949654
H	-2.19391162	-0.71537658	-2.62471790
H	-3.46903284	0.52237758	-2.55452646
H	-3.82205806	-1.32527927	3.03018483
H	-5.45615998	-0.02001359	1.65740374
H	-0.77792291	0.90049954	-1.68204201
